# Brain MRI Segmentation with Multiphase Minimal Partitioning: A Comparative Study

**DOI:** 10.1155/2007/10526

**Published:** 2007-04-19

**Authors:** Elsa D. Angelini, Ting Song, Brett D. Mensh, Andrew F. Laine

**Affiliations:** ^1^Ecole Nationale Supérieure des Télécommunications, Groupe des Ecoles des Télécommunications, CNRS UMR 5141, 75013 Paris, France; ^2^Department of Biomedical Engineering, School of Engineering and Applied Science, Columbia University, New York, NY 10027, USA; ^3^Department of Biological Psychiatry, College of Physicians and Surgeons, Columbia University, New York, NY 10032, USA

## Abstract

This paper presents the implementation and quantitative evaluation of a multiphase three-dimensional deformable model in a level set framework for automated segmentation of brain MRIs. The segmentation algorithm performs an optimal partitioning of three-dimensional data based on homogeneity measures that naturally evolves to the extraction of different tissue types in the brain. Random seed initialization was used to minimize the sensitivity of the method to initial conditions while avoiding the need for *a priori* information. This random initialization ensures robustness of the method with respect to the initialization and the minimization set up. Postprocessing corrections with morphological operators were applied to refine the details of the global segmentation method. A clinical study was performed on a database of 10 adult brain MRI volumes to compare the level set segmentation to three other methods: “idealized” intensity thresholding, fuzzy connectedness, and an expectation maximization classification using hidden Markov random fields. Quantitative evaluation of segmentation accuracy was performed with comparison to manual segmentation computing true positive and false positive volume fractions. A statistical comparison of the segmentation methods was performed through a Wilcoxon analysis of these error rates and results showed very high quality and stability of the multiphase three-dimensional level set method.

## 1. INTRODUCTION

Segmentation of three-dimensional anatomical brain images into tissue classes has applications in both clinical and research settings. Although numerous methods to segment brain MRI for extraction of cortical white matter, gray matter, and cerebrospinal fluid (CSF) have been proposed for the past two decades, little work has been done to evaluate and compare the performance of different segmentation methods on real clinical data sets, especially for CSF.

Segmenting three-dimensional anatomical brain images into tissue classes has applications in both clinical and research settings. As a clinical example, segmentation can provide volumetric quantification of cortical atrophy and thus aid in the diagnosis of degenerative diseases. These volumetric measurements apply to the research setting as well, where segmentation can also be used to define regions of interest for quantifying the physiological responses measured with fMRI or PET acquired on the same patients and coregistered with the MRI data. Clinical studies based on quantitative measurements of cortical brain structures include Alzheimer's disease [1–3], epilepsy [4], schizophrenia [5], cerebrovascular deficiency [6], and multiple sclerosis [7].

Several methods have been proposed in the literature to segment brain MRI. A good review of these methods can be found in [8] and we can distinguish two general families.

(1) Statistical methods

The first family of methods is based on *classification of brain tissues* into different classes, based on intensity values (direct values of features computed from these values). Gray values thresholding is the most intuitive classification approach [9]. One common difficulty with this method is the selection of the threshold level. Many selection approaches have been proposed based on histograms [10, 11], combination of morphological operators and region growing [12], and so forth. Derived from a statistical framework, Bayesian analysis [13, 14] is a popular classification method for brain tissue where automatic segmentation is performed with expectation maximization (EM) [4]. The freely distributed statistical parametric mapping (SPM) software tool [15] is widely used by neuroradiologists for clinical research. Additional classification methods include clustering [16, 17], fuzzy classification [18], neural networks [19], deterministic annealing [7]. Hybrid “neuro-fuzzy” methods, see [2, 20, 21], combining fuzzy logic and neural networks perform an unsupervised learning process. Another class of statistical methods is based on Markov Random Field (MRF) [22–25]. MRF model encodes spatial information through the mutual influences of neighboring voxels for class assignments. A major issue with MRF-based classification methods is the requirements for training the model and setting the MRF parameters which typically require supervised learning and *a priori* information from manual labeling or from an atlas. In this context, classification and nonuniform registration are sometimes combined together for more robustness [1, 26]. Finally, these statistical methods can be applied to multispectral MRI (i.e., MRI data of the same patient acquired with different protocols such as T1-weighted, T2-weighted, proton diffusion) using multivariate statistics. Several applications of multispectral brain MRI classification have been presented in the literature [25–27].

(2) Deformable models

The second family of segmentation methods deals with *geometric deformable models*, including active surfaces [28] and level-set-based deformable models. The level set implementation framework for surface propagation offers the advantages of easy initialization, computational efficiency, and the ability to capture deep sulcal folds. Two coupled level set surfaces were proposed by Zeng et al. [29] for cortex segmentation from 3-D MR images, assuming a constant thickness range of the cortical mantle. A combination of joint-prior shape appearance models with a level set deformable model was proposed by Yang and Duncan [30]. This method was motivated by the observation that the shapes and gray levels variations in an image had some consistent relations building a MAP shape-appearance prior model provided some configurations and context information to assist the segmentation process. The model was formulated in a level set framework rather than using landmark points for parametric shape description. Goldenberg et al. [31] proposed a geometric variational formulation for the propagation of two coupled bounding surfaces, similar to Zeng et al. [29]. The authors put forward an efficient numerical scheme for the implementation of a geodesic active surface model. Several external driving forces, derived from brain MRI, have been proposed based on image gradient [32], image intensity [33], and probability density function [28] of tissue classes. Combining classification and deformable models has been proposed by Ballester et al. [34] combining Bayesian analysis and active surface method, and Shen et al. [35] combining geometric deformable model and statistical information about the shapes of interest.

Automation of the segmentation process is critical for applications in clinical research where the number of cases to process is large and the time available for experts to analyze the data is very limited. Several of the aforementioned methods were developed in a supervised or semi-automated framework still requiring operators' intervention. Full automation can be achieved using automatic parameter tuning [10], automated initialization [12], and combination with atlas information [4].

This paper presents the comparison of the three “classical” segmentation methods: histogram-based thresholding, tissue classification based on fuzzy connectedness, and maximum-likelihood classification with hidden MRF using the FSL-FAST software tool. We also present in this paper the implementation of a new three-dimensional automated segmentation method of brain MRI using a four-phase three-dimensional active contour implemented with a level set framework. This multiphase level set framework was initially proposed by Vese and Chan [36] to simultaneously deform coupled level set functions without any prior models or shape constraints. This framework achieves a global partitioning of the image data into 2^*N*^ homogeneous areas using *N* level set curves, solely based on average gray values measures.

These four segmentation methods were applied to a set of ten clinical T1-weighted MRIs for segmentation of cortical tissues: white matter (WM), gray matter (GM), and cerebrospinal fluid (CSF). Segmentation errors are reported with comparison to manual labeling. The segmentation methods were also compared in a statistical framework to assess their relative performance and overall “quality.”

## 2. METHOD

We present in this section the four segmentation methods that were applied to ten brains T1-weighted MRI and compared together. Manually labeled data was available for this comparative study. This “ground truth” was used to optimize the parameter settings of the three “classical” segmentation methods, as detailed below.

### 2.1. Intensity thresholding

Intensity thresholding (IT) is the easiest and fastest segmentation method, often adopted for preprocessing of medical images and preregistration problems [9]. Segmentation of the three brain cortical tissues is performed via thresholding of voxel values within adjacent intervals. The position of the interval bounds was initialized as follows: we used the manually labeled data to mask the MRI data and compute the means of the three cortical tissues of interest. These mean values were used to initialize the threshold values at the two interfaces CSF/GM and GM/WM. The Tanimoto index [37] was calculated according to the manually labeled data. Finally, the simplex method [38] was applied to maximize the Tanimoto value and identify optimal interval bounds corresponding to a minimization of the segmentation error.

### 2.2. Fuzzy connectedness

Classification of homogeneous objects using fuzzy connectedness (FC) was introduced by Falcão et al. [18]. This method can be used to extract homogeneous tissues in a volume given an initial set of seed points and prior statistics defining these tissues. The source code for this method was obtained from the freely distributed insight segmentation and registration toolkit (ITK) [39] sponsored by the National Library of Medicine. Segmentation of the different tissues was performed via thresholding of the real-valued fuzzy connectedness maps. The threshold levels were also determined via minimization of the segmentation error using the simplex method [38].

### 2.3. Hidden Markov random field-expectation maximization

The FAST software tool [40] is a publicly available automated segmentation tool for brain MRI volumes. This software tool is part of the FSL comprehensive library of functional and structural brain image analysis tools, distributed by the Oxford Centre for Functional Magnetic Resonance Imaging of the Brain (FMRIB) at the University of Oxford, UK. Brain MRI volumes were segmented into three tissue types (WM, GM, and CSF) with simultaneous correction of RF bias-field. The segmentation process is based on hidden Markov random field (HMRF) models. Fitting of the HMRF model to the data was performed via maximum likelihood with expectation maximization (EM). A thorough description of the algorithm was published in [41].

### 2.4. Multiphase three-dimensional level set

The multiphase three-dimensional level set (M3DLS) segmentation method performing a minimal partitioning of the image data into piecewise constant objects, based on the Mumford-Shah functional [42], was introduced by Chan and Vese [43].

#### 2.4.1. Energy functional

This method uses a deformable model controlled by a homogeneity-based energy functional to segment piecewise constant or piecewise smooth volumetric data *u*_0_. Assuming a piecewise-constant data with an object, of value *c*_1_, and a background, of value *c*_2_, separated by the contours *C*, the proposed energy functional is defined as

(1) $$\begin{array}{l} F\left(C,c_{1},c_{2}\right)=\mu \left(\text{length}\left(C\right)\right)+\upsilon \left(\text{area}\left(\text{inside}\;C\right)\right)\\ \;\;\;\;\;\;\;\;\;\;\;\;\;\;\;\;\;\;\;\;\;\;\;\;\;\;\;\;\;+\lambda_{1}\int_{\text{inside}\;\left(C\right)}{\left|u_{0}-c_{1}\right|}^{2}d\text{Ω}\\ \;\;\;\;\;\;\;\;\;\;\;\;\;\;\;\;\;\;\;\;\;\;\;\;\;\;\;\;\;+\lambda_{2}\int_{\text{outside}\;\left(C\right)}{\left|u_{0}-c_{2}\right|}^{2}d\text{Ω}, \end{array}$$

where *μ* ≥ 0, *υ* ≥ 0, *λ*_1_, *λ*_2_> 0 are fixed parameters.

Segmentation of the data is performed via minimization of the functional *F* with respect to (*C*, *c*_1_, *c*_2_). This energy functional can be extended to the segmentation of multiple homogeneous objects in the image by using several curves {*c*_1_, *c*_2_, … , *c_i_*}. In the case of two curves the following energy functional is used:

(2) $$\begin{array}{l} F\left(c_{1},c_{2},c_{00},c_{01},c_{10},c_{11}\right)\\ \;\;\;\;\;\;=\mu_{1}\;\text{length }\left(c_{\text{1}}\right)+\mu_{2}\;\text{length }\left(c_{\text{2}}\right)\\ \;\;\;\;\;\;\;\;\;\;+\upsilon_{1}\;\text{area }\left({\text{inside c}}_{\text{1}}\right)+\upsilon_{2}\text{area }\left({\text{inside c}}_{\text{2}}\right)\\ \;\;\;\;\;\;\;\;\;\;+\lambda_{1}\int_{\text{inside}\;c_{1},\;\text{inside}\;c_{2}}\left|u_{0}-c_{11}\right|d\text{Ω}\\ \;\;\;\;\;\;\;\;\;\;+\lambda_{2}\int_{\text{inside}\;c_{1},\;\text{outside}\;c_{2}}\left|u_{0}-c_{10}\right|d\text{Ω}\\ \;\;\;\;\;\;\;\;\;\;+\lambda_{3}\int_{\text{outside}\;c_{1},\;\text{inside}\;c_{2}}\left|u_{0}-c_{01}\right|d\text{Ω}\\ \;\;\;\;\;\;\;\;\;\;+\lambda_{4}\int_{\text{outside}\;c_{1},\;\text{outside}\;c_{2}}\left|u_{0}-c_{00}\right|d\text{Ω}. \end{array}$$

**Figure 1:**
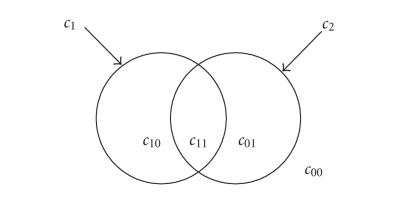
Partitioning of the image into four phases using two curves (average intensity values are designed as *c*_00_, *c*_10_, *c*_01_, *c*_11_).

The set of parameters (*λ*_1_, *λ*_2_, *λ*_3_, *λ*_4_, *μ*_1_, *ν*_1_, *μ*_2_, *ν*_2_) takes real positive values. The two closed curves (*c*_1_, *c*_2_) split the domain Ω into four phases defined by their relative positions as illustrated in Figure 1. Inside these four phases, *u*_0_ has mean intensity values (*c*_00_, *c*_01_, *c*_10_, *c*_11_).

Minimization of this energy functional deforms simultaneously the two curves and identifies four homogeneous areas defined by the intersection of the two curves.

#### 2.4.2. Level set formulation

Minimization of the functional in (1) and (2) can be performed within a level set framework. This framework, introduced by Osher and Sethian [44], provides an effective implicit representation for evolving curves and surfaces, which has found many applications in image segmentation, denoising and restoration as reviewed in [45]. In this framework, a given curve *C* (being now the boundary *∂w* of an open set *ω* ∈ Ω) is represented implicitly, as the zero level set of a scalar Lipschitz function *φ* : Ω → ℝ (called level set function), such that

(3) $$\begin{array}{l} \varphi \left(x\right)>0\;\;\;\;\text{on }\omega \text{,}\\ \varphi \left(x\right)<0\;\;\;\;\text{on }\text{Ω}\backslash \omega \text{,}\\ \varphi \left(x\right)=0\;\;\;\;\text{on }\partial \omega . \end{array}$$

The level set function *φ* is typically defined as the signed distance function of spatial points defined on Ω to the curve *C*. Once *φ* is computed, we define its associated Heaviside function *H* and Dirac function *δ* as

(4) $$\begin{array}{c} H\left(\varphi \right)=\left\{ \begin{array}{l} 0,\;\;\;\;\text{if}\;\varphi \geq 0,\\ 1,\;\;\;\;\text{if}\;\varphi <0, \end{array}\right.\\ \delta \left(\varphi \right)=\frac{d}{d\varphi }H\left(\varphi \right). \end{array}$$

Using these two functions, the different components of the functional in (1), parameterized with the contour curve *C*, can be reformulated as integrals, parameterized with the level set function *φ* and defined over the entire domain Ω.

- Length of the curve *C*:
  (5) $$\begin{array}{l} \text{Length}\left(C\right)=\text{Length}\left(\varphi =0\right)\\ \;\;\;\;\;\;\;\;\;\;\;\;\;\;\;\;\;\;\;\;\;\;\;\;\;=\int_{\text{Ω}}\left|\nabla H\left(\varphi \right)\right|d\text{Ω}=\int_{\text{Ω}}\delta \left(\varphi \right)\left|\nabla \varphi \right|d\text{Ω}. \end{array}$$
- Area inside the curve *C*:
  (6) $$\text{Area}\left(C\right)=\text{Area}\left(\varphi <0\right)=\int_{\text{Ω}}H\left(\varphi \right)\;d\text{Ω}.$$
- Homogeneity of *u*_0_ inside and outside the curve *C*:
  (7) $$\begin{array}{c} \int_{\text{inside}\left(C\right)}{\left|u_{0}-c_{1}\right|}^{2}d\text{Ω}=\int_{\text{Ω}}{\left|u_{0}-c_{1}\right|}^{2}H\left(\varphi \right)d\text{Ω},\\ \int_{\text{outside}\left(C\right)}{\left|u_{0}-c_{2}\right|}^{2}d\text{Ω}=\int_{\text{Ω}}{\left|u_{0}-c_{2}\right|}^{2}\left(1-H\left(\varphi \right)\right)d\text{Ω}. \end{array}$$

Minimization of the energy functional leads to the expression of the associated Euler-Lagrange equations for derivatives with respect to (*c*_1_, *c*_2_, *φ*). The first two derivatives, with respect to (*c*_1_, *c*_2_), lead to the following results, used for computation of the mean statistics:

(8) $$\begin{array}{c} c_{1}\left(\varphi \right)=\frac{\int_{\text{Ω}}u_{0}\left(x\right)H\left(\varphi \left(x\right)\right)d\text{Ω}}{\int_{\text{Ω}}H\left(\varphi \left(x\right)\right)d\text{Ω}},\\ c_{2}\left(\varphi \right)=\frac{\int_{\text{Ω}}u_{0}\left(x\right)\left(1-H\left(\varphi \left(x\right)\right)\right)d\text{Ω}}{\int_{\text{Ω}}\left(1-H\left(\varphi \left(x\right)\right)\right)d\text{Ω}}. \end{array}$$

The third partial derivative of *F*(*φ*, *c*_1_, *c*_2_), with respect to *φ*, leads to the following dynamic equation:

(9) $$\frac{\partial \varphi }{\partial t}=\delta_{\varepsilon }\left(\varphi \right)\left[\mu \;\text{div}\left(\frac{\nabla \varphi }{\left|\nabla \varphi \right|}\right)-\upsilon +\lambda_{1}{\left|u_{0}-c_{1}\right|}^{2}-\lambda_{2}{\left|u_{0}-c_{2}\right|}^{2}\right].$$

This segmentation framework is extended to the detection of multiple objects via the introduction of multiple level set functions {*φ*_1_, *φ*_2_, … } and the computation of mean data values in areas of constant values defined via the combination of their Heaviside functions (*H*(*φ*_1_) × *H*(*φ*_2_) × ⋯ ). In this study we implemented the segmentation functional with two level set functions generating four phases. We can introduce the binary functions *χ* (also called characteristic functions) that define the four regions:

(10) $$\begin{array}{c} \chi_{1}\left(\varphi \right)=H_{\varepsilon }\left(\varphi \right),\;\;\;\;\;\;\chi_{0}\left(\varphi \right)=\left(1-H_{\varepsilon }\left(\varphi \right)\right),\\ \chi_{11}\left(\varphi_{1},\;\varphi_{2}\right)=\chi_{1}\left(\varphi_{1}\right)\chi_{1}\left(\varphi_{2}\right),\;\;\;\;\;\;\chi_{10}\left(\varphi_{1},\;\varphi_{2}\right)=\chi_{1}\left(\varphi_{1}\right)\chi_{0}\left(\varphi_{2}\right),\\ \chi_{01}\left(\varphi_{1},\;\varphi_{2}\right)=\chi_{0}\left(\varphi_{1}\right)\chi_{1}\left(\varphi_{2}\right),\;\;\;\;\chi_{00}\left(\varphi_{1},\;\varphi_{2}\right)=\chi_{0}\left(\varphi_{1}\right)\chi_{0}\left(\varphi_{2}\right). \end{array}$$

The Euler-Lagrange equations, for the four-phase configuration, lead to the following equations defining the mean values of each phase:

(11) $$\begin{array}{c} c_{11}=\frac{\int_{\text{Ω}}u_{0}\chi_{11}\left(\varphi_{1},\;\varphi_{2}\right)d\text{Ω}}{\int_{\text{Ω}}\chi_{11}\left(\varphi_{1},\;\varphi_{2}\right)d\text{Ω}},\;\;\;\;\;\;c_{10}=\frac{\int_{\text{Ω}}u_{0}\chi_{10}\left(\varphi_{1},\;\varphi_{2}\right)d\text{Ω}}{\int_{\text{Ω}}\chi_{10}\left(\varphi_{1},\;\varphi_{2}\right)d\text{Ω}},\\ c_{01}=\frac{\int_{\text{Ω}}u_{0}\chi_{01}\left(\varphi_{1},\;\varphi_{2}\right)d\text{Ω}}{\int_{\text{Ω}}\chi_{01}\left(\varphi_{1},\;\varphi_{2}\right)d\text{Ω}},\;\;\;\;\;\;c_{00}=\frac{\int_{\text{Ω}}u_{0}\chi_{00}\left(\varphi_{1},\;\varphi_{2}\right)d\text{Ω}}{\int_{\text{Ω}}\chi_{00}\left(\varphi_{1},\;\varphi_{2}\right)d\text{Ω}}. \end{array}$$

The partial derivatives with respect to the two level set functions define the following system of equations:

(12) $$\begin{array}{c} \frac{\partial \varphi_{1}}{\partial t}=\delta \left(\varphi_{1}\right)\left\{\begin{array}{l} \mu \;\text{div }\left(\frac{\nabla \varphi_{1}}{\left|\nabla \varphi_{1}\right|}\right)-\upsilon \\ \;\;\;\;+\left(\lambda_{1}{\left(u_{0}-c_{11}\right)}^{2}-\lambda_{3}{\left(u_{0}-c_{01}\right)}^{2}\right)\chi_{1}\left(\varphi_{2}\right)\\ \;\;\;\;+\left(\lambda_{2}{\left(u_{0}-c_{10}\right)}^{2}-\lambda_{4}{\left(u_{0}-c_{00}\right)}^{2}\right)\chi_{0}\left(\varphi_{2}\right) \end{array}\right\},\\ \frac{\partial \varphi_{2}}{\partial t}=\delta \left(\varphi_{2}\right)\left\{\begin{array}{l} \mu \;\text{div }\left(\frac{\nabla \varphi_{2}}{\left|\nabla \varphi_{2}\right|}\right)-\upsilon \\ \;\;\;\;+\left(\lambda_{1}{\left(u_{0}-c_{11}\right)}^{2}-\lambda_{2}{\left(u_{0}-c_{10}\right)}^{2}\right)\chi_{1}\left(\varphi_{1}\right)\\ \;\;\;\;+\left(\lambda_{3}{\left(u_{0}-c_{01}\right)}^{2}-\lambda_{4}{\left(u_{0}-c_{00}\right)}^{2}\right)\chi_{0}\left(\varphi_{1}\right) \end{array}\right\}. \end{array}$$

We present in the next section details regarding the numerical implementation of this system.

#### 2.4.3. Numerical implementation

Segmentation was performed via iterations of the system of equations defined in (12), deforming the two level set fronts (*φ*_1_, *φ*_2_) until convergence to a stable position was reached.

The iterative scheme was organized as follows.

1. Initialize the system for time *n* = 0, with (*φ*_1_^*n*^, *φ*_2_^*n*^) defined as the distance functions from an initial set of curves.
2. For time *n* > 0, while the system is unstable,
  - compute the average values in the four phases (*c*_11_^*n*^, *c*_10_^*n*^, *c*_01_^*n*^, *c*_00_^*n*^);
  - compute the curvature and homogeneity terms of the speed, defined for each points in the spatial domain Ω;
  - compute (*φ*_1_^*n* + 1^, *φ*_2_^*n* + 1^) from (12) with the speed term (from (b)) and (*φ*_1_^*n*^, *φ*_2_^*n*^);
  - evaluate the stability of the system. Iterate at time *n* + 1 if the system is not stable.
3. When the system is stable, extract the four phases as binary volumes corresponding to (*χ*_11_^*n* + 1^, *χ*_10_^*n* + 1^, *χ*_01_^*n* + 1^, *χ*_00_^*n* + 1^).

**Figure 2:**
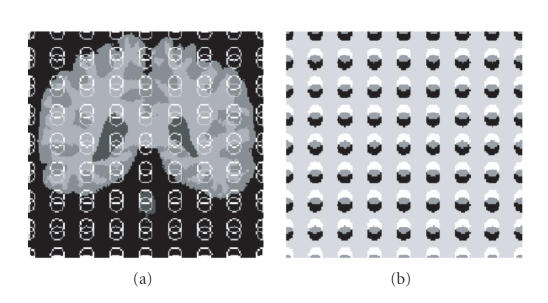
Initialization of the four-phase level set segmentation method. (a) Original MRI slice with two sets of circles initialized over the entire image. (b) Corresponding partitioning of the image domain into four phases defined by the overlap of the two level set functions obtained from the cylindrical shapes.

In our implementation, the segmentation was initialized with two level set functions defined as the distance function from two sets of initial curves. The curves were defined as 64 cylinders centered at regularly spaced seed locations across the entire data volume. The two sets of cylinders were slightly shifted from each other as illustrated in Figure 2.

Note that such initialization does not use any *a priori* information on the location of particular tissues or on the anatomy of the brain and does not require manual input by the user. As commented by Vese and Chan [36], this type of initialization also brings some robustness to the method, limiting the risks of convergence of the minimization process to local minima.

The level set algorithm was implemented with a regularized Heaviside function and a semi-implicit scheme as proposed by Vese and Chan [36] and extended to three dimensions. In this scheme, the speed term at time *n* was computed with the curvature derived semi-implicitly (using (*φ*_*i*_^*n*^, *φ*_*i*_^*n* + 1^)_*i* = 1, 2_) while the homogeneity force term was computed explicitly with (*c*_*i*_^*n*^)_*i* = 1, 2_. This semi-implicit scheme provides unconditional stability for any temporal and spatial discretization parameters. This means that we can set the time increment to an arbitrary high value to speed up the segmentation process without altering the stability of the minimization process.

### 2.5. Addressing the inhomogeneity issue

All four segmentation methods tested in this work perform a partitioning of the volumetric data into three tissue classes and a background relying on a strong assumption of tissue homogeneity for WM, GM, and CSF. The notion of homogeneity is then translated into a statistical framework (HMRF-EM, homogeneous tissue is characterized by its mean and variance), a fuzzy connectivity (FC, homogeneous tissue is characterized by high affinity measures to a prior seed point given a statistical model), or a distance measure (M3DLS, homogeneous tissue is characterized by its mean intensity value; IT, interval bounding values).

Even though homogeneity-based segmentation methods are widely used for brain MRI segmentation, it is well known that there are strong tissue inhomogeneities in MRI volumes of the following four origins: (1) biological tissues are inherently heterogeneous with internal structures and multimolecular components which contribution are integrated in the recorded MRI signal, (2) the MRI acquisition system is degraded by inherent statistical noise, (3) design of the MRI acquisition system suffers from inhomogeneity of the radio-frequency (RF) field (this phenomenon is also referred to as *RF bias-field*), (4) MRI imaging systems have limited spatial resolutions which generate *partial volume effects* (i.e., mixing of MRI signals from adjacent tissues) at tissue interface locations.

The first two sources are inherent to the modality but have minor impacts on the cortical structures that we are trying to segment. The other two sources generate artifacts that tend to become undetectable by simple visual inspection as MRI scanner technology improves, but that still constitute the major source of error and failure of homogeneity-based segmentation algorithms.

- *RF bias-field*: elaborated algorithm has been developed to correct RF bias-field [46–49]. We compared two methods commonly used and available in free-software packages. The first method was proposed by Styner et al. [47] and is available in the ITK package [50]. The second method was proposed by Ashburner and Friston [46] and is available in the FSL software package [40].
- *Partial volume effect* introduces inhomogeneities at tissue interfaces that can be modeled in a statistical framework by manipulating tissue mixture models as in [24, 51, 52]. Mixed classes are created and if necessary assigned to one of the “true” tissue classes constituting the mix. Neither the statistical HMRF-EM method nor the three other methods tested in this work included such correction and initial experiments on clinical data sets revealed misclassification of tissue labels for voxels located at tissue interfaces. We therefore derived a postprocessing sequence, based on morphological operators to correct for misclassifications at interfaces between GM and WM and between the CSF and WM or GM.

### 2.6. Postprocessing

A simple postprocessing scheme was designed to correct for pixel assignment at tissue interfaces. After the level set segmentation was completed, WM, GM, and CSF structures, corresponding to separate phases, were saved as binary volumes. These volumes were then used as masks applied to the original data and a Gaussian fit of the histograms of each phase was performed. Each phase was then characterized by the mean and standard deviation (*μ*_phase_, *σ*_phase_) of the fitted Gaussian distribution. Interface voxels were tested against the phase parameters as follows. First, the GM mask was dilated, to correct for the under segmentation of this thin structure. An interval of intensity values of [*μ*_GM_ ± 3*σ*_GM_] was selected. All new voxels, from the dilated mask, with intensity values inside this interval were included in the GM phase while voxels with intensity values outside the interval were removed from the phase and assigned to the adjacent WM phase. This process was iterated until no new points were added to the GM phase. A similar process was then applied to the CSF phase with dilation of the binary mask. An interval of intensity values of [*μ*_CSF_ ± 4*σ*_CSF_] was selected. Finally, a 3D connectivity algorithm was performed to remove isolated voxels in the three phases. This simple postprocessing approach has provided very robust performance on the ten clinical MRI cases segmented for this study.

A second postprocessing correction was needed to compare results from the different segmentation methods to manual labeling. Indeed, manual labeling did not include sulcal CSF and labeled the outer most layer of cortical brain tissue as gray matter. This was an arbitrary assignment made by the manual expert, that does not take into account partial volume effects in these voxels. A similar arbitrary classification had to be applied to the segmented data, before comparison to manual labels. This problem was especially important for the FAST HMRF-EM classification which is based on an anatomical model with sulcal CSF, leading to overestimation of the CSF on the MRI data. This oversegmentation of sulcal CSF lead to very high false positive errors when compared to manual labeling. Sulcal CSF was removed from the segmented data, after postprocessing of interface voxels, for all segmentation methods, as follows. Given the spatial resolution of the data (0.86 mm^2^ × 3 mm slice thickness), a 2-voxels dilation of the manually labeled CSF ventricles was performed in axial views, and one 1-voxel dilation was performed in the longitudinal direction. The dilated ventricle masks were applied to the segmented CSF mask and segmented voxels outside the dilated manual mask were assigned to the GM phase.

## 3. Experiments

### 3.1. Data sets

#### 3.1.1. Clinical T1-weighted MRI

We applied our segmentation to one phantom and ten T1-weighted MRI volumes acquired on healthy young volunteers. Axial slices were 1.5 mm thick with an in-plane (hyphen) resolution of 0.86 mm. These images were resliced coronally (3 mm slice thickness) and labeled via a labor-intensive (40 hours per brain) manual method in which expert raters with extensive training in neuroanatomy choose histogram thresholds on locally hand-drawn regions of interest. This labeled data was used as a “ground truth” for evaluation of the segmentation accuracy.

**Figure 3:**
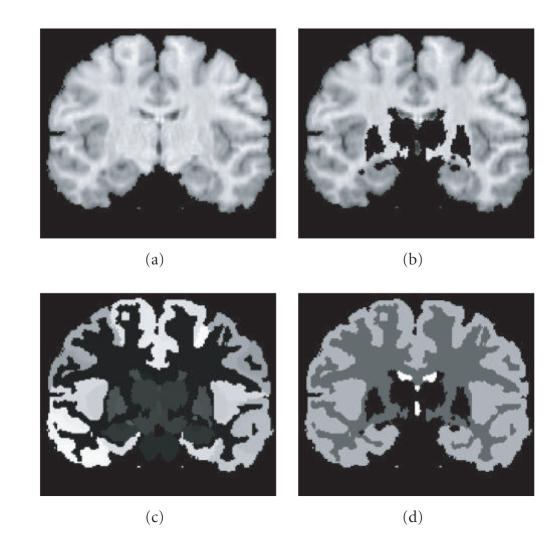
MRI brain data. (a) Original slice with cortical structures. (b) Original data with noncortical structures removed. (c) Manually labeled data on cortical structures. (d) Simplified manually labeled data used for the “ground truth.”

MRI volumes were preprocessed to remove all noncortical brain tissue by using the manually labeled data sets as binary masks. This preprocessing is illustrated in Figure 3. To determine the practicality of masking out subcortical gray matter on naïve images, we constructed from a library of labeled atlases two probabilistic atlases in which each voxel was assigned a likelihood of being made of cortical gray matter or subcortical gray matter. Less than 0.1% of the voxels in the whole brain simultaneously had over 20% chance of being made of both gray matter and subcortical gray matter. Such statistical finding confirms that one can apply a population-based method for masking out subcortical gray matter for the purpose of applying cortical segmentation methods without introducing significant errors.

#### 3.1.2. Mathematical brain phantom

A mathematical brain phantom was built from one manually labeled MRI data set to validate the performance of the multiphase level set segmentation algorithm in an ideal case. A constant intensity value was applied for each tissue corresponding to the average intensity value of the MRI data under the manual mask. This phantom data is illustrated in Figure 3.

### 3.2. Validity of homogeneity hypothesis

Prior to segmentation, we evaluated the homogeneity of the three cortical tissues: WM, GM, and CSF, computing the mean and variance statistics of the intensity distribution for each tissue within each slice. These statistical measurements were performed on axial slices across the entire data volumes for the ten MRI cases available for the study, masking the data with the manually labeled data.

**Figure 4:**
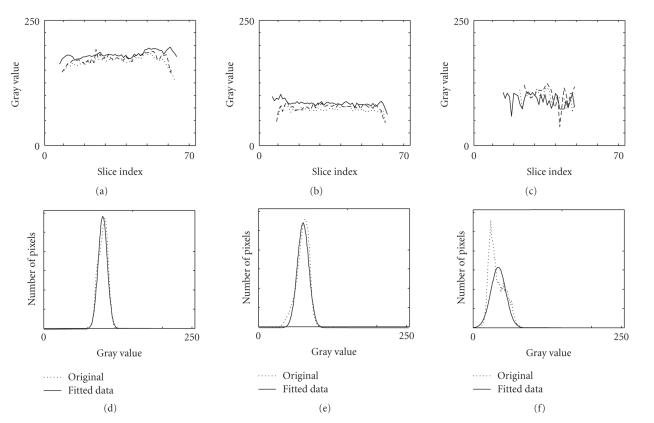
Intensity distributions and statistics for (a, d) WM, (b, e) GM, and (c, f) CSF. (a–c) Average values on consecutive slices within three MRI data sets represented with different line styles for the three MRI cases. (d–f) Fit of the volume histograms to Gaussian distributions for one MRI data set.

Results, illustrated in Figure 4 for three typical cases, showed very stable estimates of intensity mean and variance values for each tissue across the whole volume, corroborating the accuracy of the homogeneity assumption for the three tissues, and the absence of strong bias-field inhomogeneity. Lower mean intensity values on extremity slices were computed on small tissue samples (less than 10 voxels) corresponding to small anatomical structures with relatively high partial volume effects.

A Gaussian fit was performed on the histogram of the entire gray level distribution of the three brain tissues for each case. We observed that the three tissue types have well-separated average values suggesting that global homogeneity measurements could separate tissue types for each patient. Nevertheless, the agreement between the volume histograms and the fitted Gaussian distribution was evaluated with a chi-squared test. Results for the different tissues did not show a systematic agreement between the data and the Gaussian fit, at level 0.05, except for the gray matter. Therefore, despite reasonable homogeneity, we need further investigation before being able to introduce additional constraints based on *a priori* Gaussian statistics to the method as proposed, for example, by Baillard et al. [28].

### 3.3. Quantitative evaluation of segmentation performance

**Figure 5:**
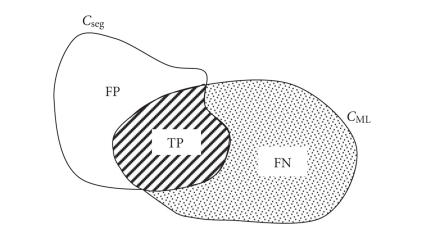
Quantitative measures of segmentation accuracy with volume fraction overlap and differences.

Segmentation errors were measured using the recent methodology from Udupa et al. [39] for comparison of segmentation methods. Accuracy of the object contours obtained with the proposed segmentation method, referred to as *C*_Seg_, was evaluated by comparing the results to our “ground truth” segmentation of each object, using manually labeled contours, referred to as *C*_ML_. The overlap and difference between the two contours were measured via counting the true positive (*TP*), false positive (*FP*), and false negative (*FN*) voxels as illustrated in Figure 5. These quantities are reported as volume fractions (*VF*) of the true delineated object volume *C*_ML_ as follows.

- FNVF = fraction of tissue defined in *C*_ML_ that was missed by the segmentation method *C*_Seg_.
- FPVF = fraction of tissue defined in *C*_Seg_ falsely identified by the segmentation method.
- TPVF = fraction of the total amount of tissue in the segmentation *C*_Seg_ which overlaps with the true object *C*_ML_.

We point out here that TPVF + FNVF = 1, so that we only need to observe TPVF and FPVF measures to evaluate the segmentation method.

## 4. RESULTS

### 4.1. Evaluation of bias-field correction

In order to evaluate the two bias-field correction methods and their effects on the homogeneity of the tissues, we applied the intensity thresholding (IT) segmentation before and after bias-field correction, providing three groups of results for the ten MRI cases: segmentation of original data (RAW), segmentation of corrected data with the ITK software tool (ITK), and segmentation of corrected data with the FSL software tool (FSL). The IT segmentation method was selected as it only relies on good separation of the tissue range of intensities, without any additional constraint or model. The FSL bias-field correction did not need any parameter setting, and the corrected data was generated along with the segmentation via HMRF-EM. The bias-field correction with the ITK toolkit used the following parameters: mean and standard deviation of the three tissue classes were calculated from the “ground truth,” the input and output mask with nonbrain tissues removed were also generated from the “ground truth,” the degree of the method was set to 2, growing parameter was set to 1.05, shrinking parameter was set to 0.9 (suggested values), and a maximum of 2000 iterations were performed with an initial step size of 1.02.

**Figure 6:**
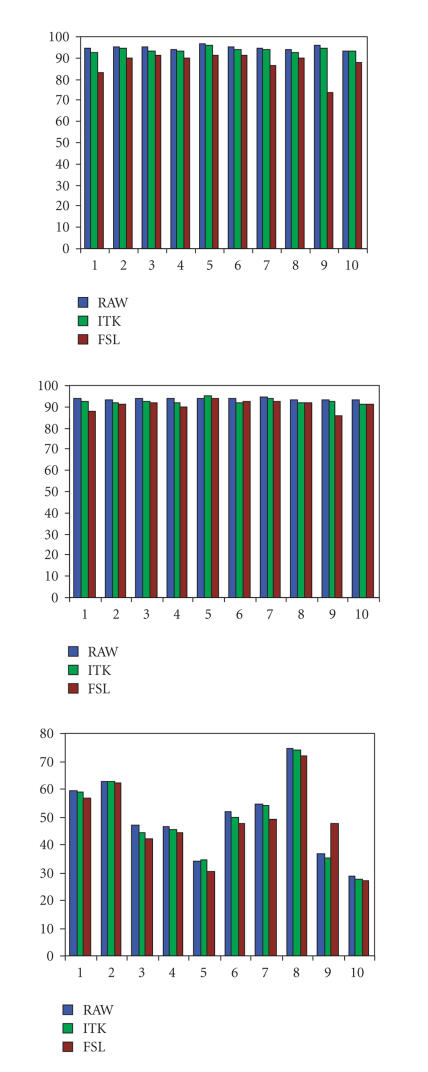
Evaluation of bias-field correction on IT segmentation on the 10 MRI cases. Bar plots of TPVF of (top) GM, (middle) WM, (bottom) CSF.

Effects of bias-field correction on IT segmentation are illustrated with bar plots of TPVF, in Figure 6.

We observed that the bias-field correction methods did not improve and even degraded the TPVF accuracy of the IT segmentation method. The ITK method provided similar accuracy for all cases and all tissues while the FSL method tended to degrade TP performance, except for the CSF on one MRI case. Based on homogeneity results presented in Figure 4, these results and the difficulty involved with parameter settings of the bias-field correction methods, we decided to exclude bias-field correction for this study.

### 4.2. Comparison of segmentation methods

The level set segmentation was initialized for the phantom and MRI clinical cases with two sets of regularly spaced cylinders as illustrated in Figure 2. A stable behavior of the four phases was observed after 10 iterations.

#### 4.2.1. Brain phantom

Segmentation of the brain phantom was performed with three phases corresponding to the cortical tissues and the 4th phase to the background. In Table 1, we observed almost perfect segmentation of the brain structures, demonstrating the inherent ability of the multiphase level set framework, initialized with small regular cylinders, to the following.

- Identify the four homogeneous phases.
- Extract highly convoluted surfaces.
- Perform topology splitting and merging of the evolving front, enabling the identification of separate structures within a single phase (such as the three ventricles for the CSF) that exist as different spatial objects.

Computation of homogeneity-based speed terms for phases with small structures, such as the CSF, suffered from higher estimation inaccuracy. In the absence of a curvature term, the homogeneity-based speed terms generate a *k*-means classification of the image data with poor quality extraction of these small structures. In this context, the curvature term of the deformable model plays a critical role in the M3DLS to preserve small structures shapes and sizes. Partial volume effect was not simulated in the phantom and therefore no postprocessing was applied.

We used this phantom to tune the parameters of the segmentation method set to

(13) $$\begin{array}{c} \lambda_{1}=\lambda_{2}=\lambda_{3}=\lambda_{4}=0.01,\;\;\;\;\;\;\upsilon =0,\\ \mu =4.10^{-8}\times \text{​}\;\text{Volume}\_\text{size}/\text{Diagonal}\_\text{distance},\\ \Delta t=10^{4},\;\;\;\;\;\;\Delta x=\Delta y=\Delta z=1. \end{array}$$

The parameter associated with the curvature term is defined proportional to the data volume size (*Volume_size*) and inversely proportional to the diagonal distance of the volume data (*Diagonal_distance*). By doing so, we consider this diagonal distance as the unitary distance of our domain of definition Ω. Setting the constant speed term *υ* to zero eliminates the use of a constant inflating force on the model. This type of constant force should be used with caution as it can override the homogeneity-based speed term when driving the deformable contour and force it to move in only one direction (i.e., constant inflation or deflation).

#### 4.2.2. Clinical brain MRI

**Figure 7:**
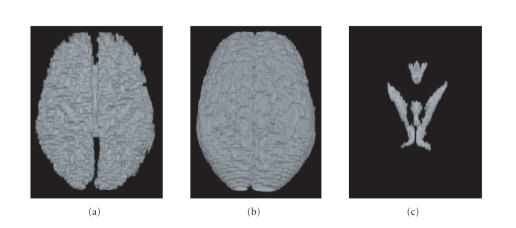
Segmentation of one MRI data set. Three-dimensional rendering of segmented volumes for (a) WM, (b) GM, and (c) CSF.

We present in Figure 7 a three-dimensional rendering of each segmented structure for one MRI clinical case segmented for this study.

Visual rendering of the three cortical structures confirmed the overall high performance of the multiphase segmentation method to extract homogenous objects that correspond to distinct anatomical tissues.

The segmentation method was able to handle multiple challenges without any *a priori* information or shape constraints that include the extraction of highly convoluted white matter surfaces, the extraction of separate ventricular structures for the CSF, and handling of different volume sizes of the three structures in a simultaneous segmentation scheme.

**Figure 8:**
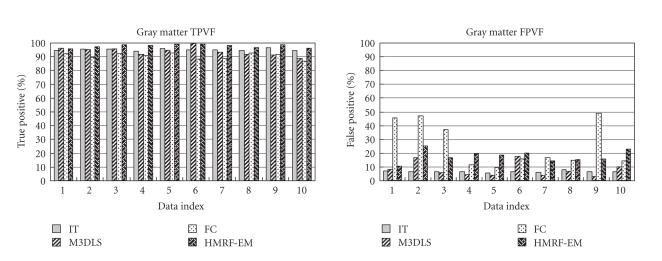
TPVF and FPVF errors for segmentation of GM.

**Figure 9:**
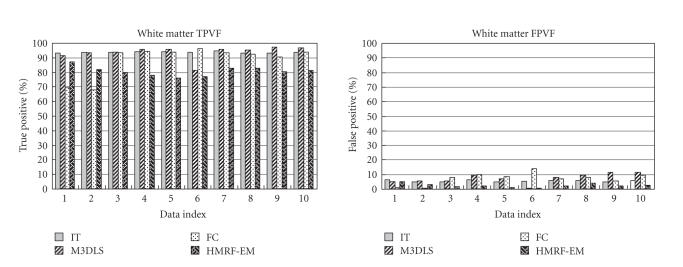
TPVF and FPVF errors for segmentation of WM.

**Figure 10:**
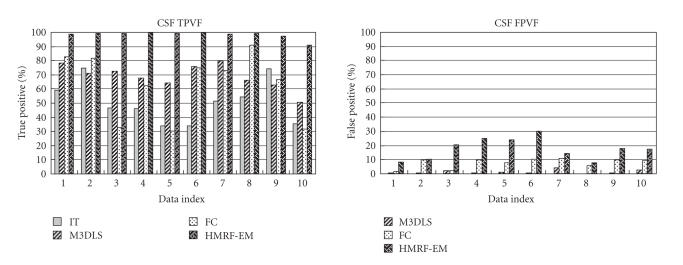
TPVF and FPVF errors for segmentation of CSF.

Accuracy of the object contours obtained with the proposed multiphase level set deformable model and the three other segmentation methods was evaluated by comparing segmentation results to the manual ground truth. Bar plots of error measurements for the segmentation of the ten clinical cases and average errors are reported in Figures 8, 9, and 10. Because TPVF and FNVF are both relative measures with respect to the ground truth volume, we have TPVF + FNVF = 1. Therefore, we only generated plots for TPVF and FPVF.

We can make the following observations based on the results presented in these graphs for *segmentation of GM*, characterized as a thin structure with large surface area: HMRF generates the highest TPVF and FC the lowest. IT and M3DSL perform well (TPVF > 85%) and stably over the 10 cases. FC generates over-segmentation of the GM with high FPVF (up to 48%), followed by HMRF-EM (up to 25%). M3DSL (FPVF < 18%) generates low and stable over-segmentation errors, and IT (FPVF < 10%) does not generate this type of error. Overall, IT provides the best performance for TPVF and FPVF, followed by M3DLS, with good performance. FC showed lower sensitivity and HMRF-EM lower selectivity.

We can make the following observations based on the results presented in these graphs for *segmentation of WM* (characterized as a large structure): IT and M3DSL generated high stable and similar TPVF (TPVF > 90%), FC generated low TPVF on 2 cases, while HMRF-EM systematically generated lower TPVF. All segmentation methods generated very low over-segmentation errors. M3DLS and FC showed FPVF errors up to 10%. Overall, IT and M3DLS showed the highest sensitivity with very good specificity.

We can make the following observations based on the results presented in these graphs for *segmentation of the CSF* (characterized as small disconnected structures): HMRF-EM was the only segmentation method to show very high TPVF (> 90%). All other three methods generated variable errors over the 10 cases with M3DLS generating the smaller range of errors (50% < *TPVF* < 80%). In particular, IT performed very poorly, certainly due to the absence of shape constraint. HMRF showed large over-segmentation errors (above 10% and up to 30%), while M3DSL showed very low FPVF errors (< 5%). Overall, HMRF-EM showed perfect sensitivity along with poor selectivity (i.e., over segmented the CSF) and M3DLS showed the best tradeoff of sensitivity versus specificity.

The four segmentation methods were compared statistically using a characteristic index of their performance. For this task, the Tanimoto index (TI) was selected [37]. This index is a quantitative parameter used to evaluate the segmentation results and is defined as

(14) $$\text{TI=}\frac{\text{TPVF}}{\text{1+FPVF}}.$$

**Figure 11:**
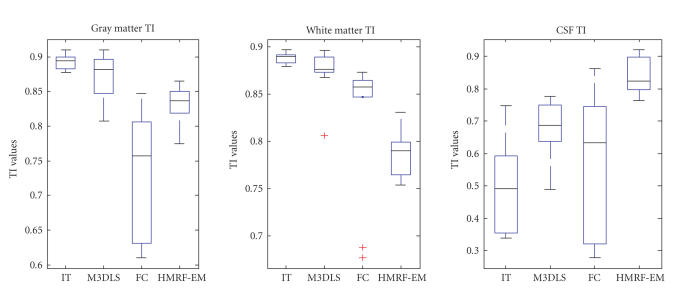
Box plot of TI values, over the four segmentation methods (IT, M3DLS, FC, HMRF-EM) for the 10 clinical cases. for GM, WM and CSF.

Because TI populations do not follow a normal distribution, a nonparametric analysis was performed for the four methods over the 10 segmented cases, measuring the differences of the TI indexes. Small *p* values (below 0.05) indicate a significant statistical difference between the methods [53]. Distributions of the TI index over the 10 cases for each method are plotted in Figure 11.

Box limits are placed at the lower and upper quartile values. The median value is indicated by a line inside each box. Whiskers are lines extending from each end of the box to show the extent of the rest of the data (1.5 interquartile ranges). Outliers, identified with red crosses, are data with values beyond the ends of the whiskers. If there is no data outside the whisker, a dot is placed at the bottom whisker.

These results illustrate graphically the average performance and the variability of performance of individual segmentation methods. We observed that M3DLS and HMRF-EM generated the most stable performance over the three tissue types, with mean performance higher with M3DLS for WM and GM.

Evaluation of the statistical differences of results from the four segmentation methods was performed with a Wilcoxon signed rank test for paired data. Significance values for the three tissue types for each pair of segmentation methods are reported in Table 2.

From these results we see the following.

1. For GM, the two best methods, M3DSL and IT were not statistically different.
2. For WM, the two best methods, M3DSL and IT were statistically different. The two weakest methods, FC and HMRF-EM were not statistically different.
3. For CSF, the two best methods, M3DSL and HMRF-EM were statistically different. On the other hand, FC was not statistically different from M3DSL and IT. Therefore, for CSF, HMRF-EM is superior to the three other methods. Compared with IT and FC, M3DLS has a higher mean, and smaller variance, which shows better performance and more stability.

*Specific problems for segmentation of the CSF phase*

**Figure 12:**
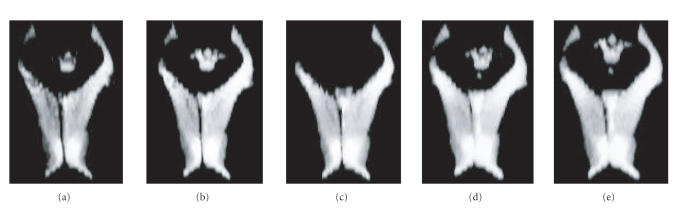
CSF segmentation. Three-dimensional rendering of the ventricles extracted with: (a) IT, (b) M3DLS, (c) FC, (d) HMRF-EM, and (e) manual labeling.

As observed in the error plots and illustrated in Figure 11, all methods except HMRF-EM performed significantly poorly on CSF than on WM and GM, corresponding to under segmentation of the ventricles, whose pixels were assigned to white matter. On the other hand, the HMRF-EM segmentation was very sensitive but provided poor specificity. Very low resolution at the ventricle borders explains in part this result. In addition, manual labeling of the MRI data for the ventricle can also bear some error as localizations of its borders are difficult even for an expert performing manual tracing. We illustrated in Figure 12 CSF segmentation with the different methods with a three-dimensional rendering of the two lateral ventricles and a section of the third ventricle.

We also recall that manual labeling is only used as a method of reference, and does not provide a real “ground truth” to the segmentation problem. In that context, Kikinis et al. reported in [54] a variation in volumetric measurements between manual observers in the order of 15% for WM, GM, and CSF.

## 5. DISCUSSION

This study used a 3D implementation of a segmentation framework initially proposed by Chan and Vese [43]. A single illustration of the ability of the method to segment a brain MRI slice was illustrated in their paper. No group has previously implemented and tested this method on a whole-brain MRI dataset, with fixed parameter settings, which is a critical aspect for the demonstration of the ability of such a segmentation approach to be an efficient clinical tool. We chose to use a whole-brain MRI data set in order to establish a benchmark of the performance of existing brain MRI segmentation tools, as well as to develop a new automated and robust segmentation tool that would alleviate the need for *a priori* knowledge such as tissue statistics and the need for manual initialization.

We can compare our segmentation error to results reported by Zeng et al. [29] and Niessen et al. [55]. In Zeng et al. [29], the authors tested their algorithm for the segmentation of frontal lobes on seven high-resolution MRI datasets from a randomly chosen subset of young autistic and control adult subjects. They ran a coupled-surfaces level set algorithm to isolate the brain tissue and segment the cortex. The average TPVF and FPVF for the cortical GM in the frontal lobe were 86.7% and 20.8%. In Niessen et al. [55], a “hyperstack” segmentation method, based on multiscale pixel classification, was tested for 3D brain MRI segmentation. A supervised segmentation framework with manual postediting was applied to a probabilistic brain phantom for estimation of segmentation error. First, a binary segmentation of the brain phantom was performed to evaluate the minimal segmentation error due to partial volume effects. The study reported a volume fraction of misclassified pixels (FPVF + FNVF) around 20% for WM, GM, and CSF. “Hyperstack” segmentation was applied with and without a probabilistic framework. Optimal (FPVF + FNVF) errors were obtained with the probabilistic version reporting 10% for WM, 21% for GM, and 25% for CSF.

In our case we proposed a fully automated segmentation method with no *a priori* information, leading to the following conclusions.

- For GM, we showed that the M3DLS method was statistically equivalent to the idealized IT with average (FPVF + FNVF) equal to 11.39% for IT and 14.23% for M3DLS.
- For WM, M3DLS produced an average (FPVF + FNVF) equal to 13.56%, superior to HMRF-EM with 21.64% and FC methods with 19.57%.
- For CSF, M3DLS, which produced an average (FPVF + FNVF) equal to 32.56%, was found statistically superior to FC with (FPVF + FNVF) equal to 45.0% and 48.92% for IT.

The random initialization ensured robustness of the method to variation of user expertise and biased or erroneous input information that could be influenced by variation in image quality or user expertise. The method is automated, given that the set of parameter values selected on the brain phantom is suited for the clinical data.

Comparing the M3DLS method to the other ones, we see automation as a major advantage compared to the supervision required by FC segmentation and stability of performance over clinical cases and tissue types as a major advantage over the HMRF-EM method. The IT was set up with ideal parameters and cannot be considered as a potential segmentation method. It was just proposed to test ideal clustering based on voxel intensity.

*Noncortical structures*

Using two simultaneous level set functions limits the segmentation process to the extraction of four homogeneous phases, corresponding to four tissue types at most. Segmentation of several other noncortical brain structures including the thalamus, the caudate, the putamen, the palladium, the hippocampus, and the amygdale is important for detection of neurological diseases such as schizophrenia or Alzheimer's disease. As shown in a recent study for manual labeling of whole brain MRI data [56], these structures have greatly overlapping grayscale distributions which explains the failure of simple thresholding methods to perform efficient extraction of these individual more subtle structures. On the other hand, as long as the histograms show reasonable compactness (with a small standard deviation), the homogeneity-based proposed method should be capable of segmenting them into separate phases. For the present study, we only focused on cortical structures. Subcortical structures were therefore removed prior to segmentation using the manually labeled data to mask them, as illustrated in Figure 3. For future applications of the method to data sets without the benefit of manually labeled data, we can either use the labeling software used by clinicians to remove these structures or use an in-house “intelligent manual segmentation tool” such as the one designed by Barrett et al. [57].

## 6. CONCLUSION

This paper presented a novel clinical application and quantitative evaluation of a recently introduced multiphase level set segmentation algorithm using T1-weighted brain MRIs. The segmentation algorithm performed an optimal partitioning of a three-dimensional data set based on homogeneity measures that naturally evolved toward the extraction of different tissue types and cortical structures in the brain. Experimentation on ten MRI brain data sets showed that this optimal partitioning successfully identified regions that accurately matched WM, GM, and CSF areas. This suggests that by combining the segmentation results with fiducial anatomical seed points, the method could accurately extract individual structures from these tissues. Random initialization was used to speed up the numerical calculation and avoid the need for *a priori* information input. A regular initial partitioning of the data added some robustness to the presence of local minima during the optimization process. Comparison to three other segmentation methods was performed with individual assessment of segmentation performance, statistical comparison of the performance, and evaluation of the statistical difference between the methods. Results showed very high quality and stability of the M3DLS method.

Future work will include postprocessing of the segmented volumes to extract individual structures such as the ventricles as well as incorporation of available coregistered T2-weighted MRI and PET data to improve the segmentation performance by running the algorithm on vectorial-type data. Such extension of the method has been proposed for color images but never for multimodality clinical data.

## Figures and Tables

**Table 1:** Error measurements for the segmentation of the brain phantom.

Tissue	FPVF	TPVF

WM	0	100%
GM	0.2%	100%
CSF	0%	84.6%

**Table 2:** Significance values for GM, WM, and CSF for 6 pairs of segmentation methods.

*p*	GM	WM	CSF

IT—M3DSL	0.103	0.005	0.017
IT—FC	0.005	0.005	0.333
IT—HMRF-EM	0.005	0.005	0.005
M3DSL—FC	0.005	0.047	0.114
M3DSL—HMRF-EM	0.005	0.005	0.005
FC—HMRF-EM	0.009	0.386	0.005
